# Application of Near-Infrared Reflectance Spectroscopy for Predicting Damage Severity in a Diverse Panel of *Tectona grandis* Caused by *Ceratocystis fimbriata*

**DOI:** 10.3390/plants12142734

**Published:** 2023-07-23

**Authors:** Isabela Vera dos Anjos, Mohsin Ali, Freddy Mora-Poblete, Kelly Lana Araujo, Thiago Alexandre Santana Gilio, Leonarda Grillo Neves

**Affiliations:** 1 Doctoral Program in Biotechnology and Biodiversity, Pro-Midwest Network, Cáceres 78210-778, Brazil; iveradosanjos@hotmail.com (I.V.d.A.); kellylana@unemat.br (K.L.A.); thiago.gilio@ufmt.br (T.A.S.G.); leonardaneves@unemat.br (L.G.N.); 2 Institute of Biological Sciences, University of Talca, Talca 3460000, Chile; mohsin.ali@utalca.cl; 3 Institute of Agricultural and Environmental Sciences, Federal University of Mato Grosso, Sinop 78550-728, Brazil; 4 Faculty of Agricultural and Biological Sciences, State University of Mato Grosso, Cáceres 78210-778, Brazil

**Keywords:** ceratocystis wilt, partial least squares regression, spectral reflectance indices, severity of infection, wavelengths

## Abstract

*Tectona grandis* Linn., also known as teak, is a highly valued species with adaptability to a wide range of climatic conditions and high tolerance to soil variations, making it an attractive option for both commercial and conservation purposes. In this sense, the classification of cultivated teak genotypes is crucial for both breeding programs and conservation efforts. This study examined the relationship between traits related to damage in the stem of teak plants caused by *Ceratocystis fimbriata* (a soil-borne pathogen that negatively impacts the productivity of teak plantations) and the spectral reflectance of 110 diverse clones, using near-infrared spectroscopy (NIRS) data and partial least squares regression (PLSR) analysis. Cross-validation models had R^2^ = 0.894 (ratio of standard error of prediction to standard deviation: RPD = 3.1), R^2^ = 0.883 (RPD = 2.7), and R^2^ = 0.893 (RPD = 2.8) for predicting stem lesion area, lesion length, and severity of infection, respectively. Teak genotypes (clones) can benefit from the creation of a calibration model utilizing NIRS-generated data paired with PLSR, which can effectively screen the magnitude of damage caused by the fungus. Overall, while the study provides valuable information for teak breeding and conservation efforts, a long-term perspective would be essential to evaluate the sustainability of teak genotypes over various growth stages and under continuous pathogen pressure.

## 1. Introduction

*Tectona grandis* Linn. (Family: Lamiaceae), also known as teak, is a highly valued tree species due to its durable and resistant wood, making it a popular choice for outdoor furniture, flooring, and shipbuilding [1]. Teak trees are also used for reforestation and agroforestry [2]. The growing demand for teak wood, along with an increase in planted area, has led to renewed interest in the genetic management of this species among foresters and breeders [3]. In this sense, the classification of cultivated teak genotypes is crucial for both breeding programs and conservation efforts, including the identification and protection of unique genetic sources, essential for the preservation of the species and its commercial success [4]. Teak is known for its adaptability to a wide range of climatic conditions, from hot and dry deserts to extremely wet climates, and it can thrive in areas with annual rainfall of less than 500 mm and more than 5000 mm per year [2], as well as in temperature ranges from 2 °C to 48 °C [5]. In addition to its adaptability to a wide range of climatic conditions, teak also has a high tolerance to soil variations and can grow in a variety of soil types, including well-drained sandy, loamy, and clay soils [2]. Studies have also found that different teak genotypes have high levels of resistance to insect pests and diseases, making it a suitable option for sustainable forestry practices [6]. Overall, the versatility and resilience of teak make it an attractive option for both commercial and conservation purposes.

In Brazil, some of the most sought-after tree species include eucalypts, pine, black wattle, rubber tree, parica, Populus, and teak. Among these species, the area under teak plantation is increasing in Brazil, in order to obtain high-quality wood and to target the international wood market for export purposes [7]. Teak plantations are mainly found in the Midwest region of Brazil, with the state having the largest teak plantation of about 50,000 hectares, due to the favorable climatic conditions for future expansion. According to the Köppen climate classification, the climate in this area is tropical wet–dry [8]. However, an unfavorable climate can not only negatively impact the productivity of teak plantations, but also affect the growing season and plant growth in the following season [9]. 

**Figure 1:**
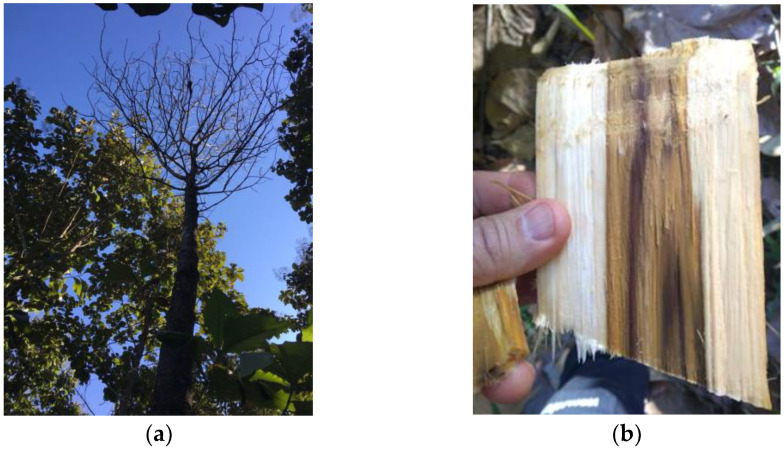
Symptoms caused by *Ceratocystis fimbriata*. (**a**) Wilt and death of a teak tree in Brazil due to infection. (**b**) Infected stem of a teak tree showing visible symptoms.

In 2009, reports emerged of teak plants displaying wilt symptoms in the Cáceres region of the state of Mato Grosso (MT), Brazil, for unknown reasons. Pathogenicity tests were conducted to investigate the symptoms, and Firmino et al. (2012) [10] confirmed the presence of the soil-borne pathogen *Ceratocystis fimbriata* as the cause of the wilt in this region. This pathogen has been found to have a wide range of native and exotic hosts in Brazil [11,12,13,14]. Wilt disease is among the factors that negatively impact the production potential, durability, and quality of teak wood. The emergence of pathogens such as *C. fimbriata*, which causes wilt, can be attributed to multiple factors [15,16]. This fungus has been reported as lethal for various other important fruits and agronomic crops, as well as forest plantations in Brazil [10,17,18]. Ceratocystis wilt is currently one of the major limitations and significant threats to teak plantations in Brazil and South America. Infected teak plants exhibit visible symptoms such as wood lesions, wilting, and dry pointers, which ultimately lead to the death of the entire plant [19,20,21], as shown in Figure 1. The fungus is primarily spread through wounds in the plant, such as those caused by pruning or insect damage [14]. In this context, the objectives of this study were to evaluate traits related to the magnitude of damage caused by the fungus *Ceratocystis fimbriata* in a diverse panel of teak clones, and to examine the relationship between these traits and the spectral reflectance of the leaves. We present a novel approach to evaluate traits related to damage caused by the fungus *C. fimbriata* in teak clones. Our analysis incorporates near-infrared spectroscopy (NIRS) data and partial least squares regression (PLSR) to establish cross-validation models for predicting stem lesion area, lesion length, and severity of infection. This analytical method development using NIRS and PLSR offers a robust and efficient way to screen the magnitude of damage caused by the pathogen, providing valuable insights for teak breeding and conservation efforts.

## 2. Results and Discussion

The present study provides valuable insights into the varying resistance of different teak clones to *Ceratocystis fimbriata*, a fungus that poses a significant threat to teak plantations in Brazil and South America, and highlights the potential of using spectral reflectance of leaves as a tool for predicting the magnitude of damage caused by the fungus. Table 1 shows the results of the statistical analysis conducted on growth-related traits, including plant height, stem basal diameter, and apex diameter, as well as phenotypic traits related to the magnitude of damage caused by the pathogen *Ceratocystis fimbriata* in a diverse panel of *Tectona grandis*. The heritability values indicate the extent to which the observed variation in each trait can be attributed to genetic factors. Furthermore, the significant effects of genotype on these traits confirm that the genetic diversity present in the teak panel is a determining factor in the variability of these traits. The significant differences observed between the genotypes for each trait suggest that these traits could be potentially used in breeding programs to select for improved resistance to Ceratocystis wilt, as well as for increased early growth [7,21].

Table 2 shows the descriptive statistics and the number of samples for the calibration and validation used in this study. In this study, we developed partial least squares regression (PLSR) models to predict the severity of Ceratocystis wilt in teak plants using both raw spectral data (range of 895–2521 nm) and spectral reflectance indices (SRIs) calculated from 108 spectral bands (Supplementary Materials Table S1). The calibration equations were created using a training dataset of 84 samples, and the models were cross-validated using an internally stratified testing dataset. This approach allowed us to assess the performance of the models and select the most accurate one for predicting the severity of Ceratocystis wilt.

Table 3 presents the performance of the PLS regression models in predicting traits related to fungal infection damage in teak clones, evaluated by different metrics. The results showed that the best performing PLS cross-validation model was obtained for the trait Lesion Area, using NIR data. The model had a coefficient of determination of R^2^ (val) = 0.894, indicating that 89.4% of the variation in the data was explained by the model. The model also had a ratio of standard error of prediction to standard deviation (RPD) of 3.1, indicating a good precision of the model, and a low standard error of prediction (SEP) of 0.041, indicating a good accuracy of the model.

On the other hand, the model with the lowest coefficient of determination was the one adjusted for the Lesion Length trait with a value of R^2^ = 0.745, with RPD = 2.7, with the SRI data as predictors. Overall, the traits predicted using NIRS data combined with PLSR were superior to those predicted by SRI data, indicating that spectral signatures are efficient at predicting the severity of damage by the fungus at the leaf level. The results of the study suggest that different teak clones exhibit varying levels of resistance to the fungus. Additionally, we confirmed that spectral reflectance can be used as a non-destructive, rapid, and cost-effective tool to identify resistant clones to a specific pathogen [22]. For instance, Ali et al. (2019) [23] provides an overview of non-destructive techniques used for plant disease detection. The authors emphasize the need for early detection and diagnosis of plant diseases, which can reduce the use of pesticides and prevent crop losses. The article discusses various non-destructive techniques, including spectral reflectance (i.e., NIR spectroscopy).

**Figure 2:**
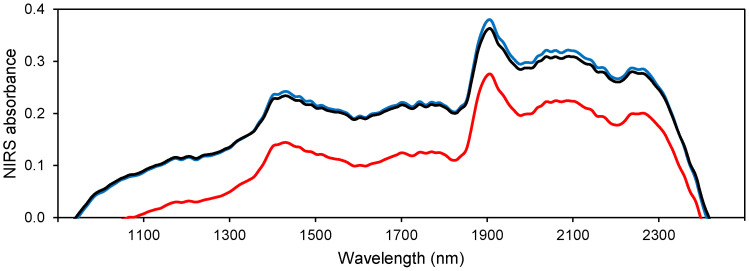
Average spectral curves of various genotypes categorized based on their susceptibility or resistance to Ceratocystis wilt. The spectral reflectance of the more resistant group is depicted by the blue line, while the red line represents the average spectral reflectance of the susceptible group. The black line represents the mean spectral reflectance of non-infected plants, serving as a reference point.

Figure 2 displays the average spectral curves for two distinct groups comprising four clones in each group, which differ in their susceptibility and resistance to Ceratocystis wilt in the experiment (*p* < 0.01). The relatively resistant group had an average severity of 10.4%, whereas the susceptible group had a mean severity of 44.6%. This variation can be utilized for detecting relatively resistant genotypes using NIRS data. This study confirms our hypothesis and provides new insights for further research on teak genotypes’ response to the *C. fimbriata* fungus and the development of resistant teak genotypes. With the lack of registered fungicides for efficient control of Ceratocystis wilt in teak, there is a crucial need to adopt alternative disease management practices like the spectral-based approach used in this research. A previous study by Oliveira et al. (2021) [21] found significant variation in the resistance and susceptibility of 37 teak clones to the fungus, with five clones exhibiting high resistance, although none of the genotypes studied was immune. The study emphasizes the importance of selecting resistant clones (regarding the damage of the stem) for controlling wilt disease in teak, increasing plantation productivity.

The combination of NIRS data with the PLSR approach has proven to be a robust and highly effective method for predicting complex response variables. In this study, we conducted a thorough evaluation of essential metrics, including SECV, SEP, and RPD, to assess the predictive capacity of spectral reflectance data for the investigated traits. The results of these statistical metrics indicate that the spectral reflectance data can reliably predict the traits under study, aligning with the acceptance criteria established in a previous study conducted by Renner et al. in 2020 [24]. PLSR was designed to handle situations where there are many possibly correlated predictor variables and relatively few samples, a situation that is common in chemistry, where developments in spectroscopy since the 1970s have revolutionized chemical analysis [25]. Notably, in the current study, the PLSR models based on spectral reflectance correctly predicted the magnitude of damage in the stem in about 90% of individual teak clones. Moreover, to assess the stability and generalization ability of these models, the leave-one-out cross-validation (LOOCV) method was employed. LOOCV is a particularly useful technique when working with limited datasets, as it allows each individual teak clone’s data to serve both as a training set and as an independent testing set. This method ensures that every observation is tested exactly once, mitigating the risk of over-fitting, and providing a more robust evaluation of the model’s performance on unseen data [26,27,28]. 

The field of NIR spectroscopy, with its highly overlapping lines and difficult-to-interpret overtones, would not have existed but for a method to obtain quantitative information from the spectra [29,30,31,32]. This reliable, cost-effective, fast, and versatile analytical tool has the potential to assess resistant teak clones to *C. fimbriata* and pave the way towards applied teak improvement programs for quality timber production [33]. Overall, several studies have demonstrated the potential of NIRS data and PLSR in various applications, including identifying resistant plant genotypes to pathogens and assessing soil properties and nutrient content.

## 3. Materials and Methods

### 3.1. Plant Material

This study was conducted using teak (*Tectona grandis*) clones from the germplasm maintained by the Genetic Breeding Laboratory (GBL) of the State University of Mato Grosso (UNEMAT), which represent an important source of variability. The field experiment was conducted in a randomized complete block design (RCBD), with three blocks, 110 treatments (genotypes/clones) and three repetitions per block. The study was conducted in the experimental station of UNEMAT in a greenhouse (under 70% shading), from December 2019 to June 2020.

### 3.2. Fungal Strain and Inoculation

The inoculum of *C. fimbriata* was the accession LMGVCF 22 from the GBL. The fungus was grown in MYEA medium (2% malt extract, 0.2% yeast extract, 2% agar) in Petri dishes. Plates were incubated for 10 to 15 days until complete colonization [34]. All the seedlings were cultivated from cuttings, so after transplanting, we waited 12 weeks (84 days) for inoculation. During this acclimatization process and the entire experiment period, the plants were kept under a 70%-shade net. After acclimatization, the seedlings were inoculated according to the mycelium disk methodology [35] with adaptations. For this, a circular wound was made at the base of the stem of each plant with the aid of a cork punch; in this wound was then inserted a disc of MYEA culture medium colonized by *C. fimbriata*.

### 3.3. Quantification of Fungal Damage in Teak Stems

To quantify the magnitude of damage caused by fungal colonization in vascular tissues, we vertically cut and photographed each plant 120 days after inoculation. Using ImageJ software [36], we processed the photographs of the plant stems to accurately measure the area of the lesion, providing an objective measurement of the severity of the disease. Subsequently, we considered the following traits to quantify the damage to seedlings: Severity (S; in %), Lesion Area (LA; in cm^2^), and Lesion Length (LL; in cm). Severity was determined by using the total plant height in centimeters (AT) and the compression of the lesion in centimeters (CL) by the methodology proposed by [13,21], using the formula (CLx100)/AT.

To complete Koch’s postulate, we collected segments of the symptomatic tissue from each genotype for re-isolation using the carrot bait method, as described by Oliveira et al. [21]. These measures and the re-isolation method allowed us to accurately evaluate the resistance of each teak genotype to *C. fimbriata*. 

### 3.4. Spectral Reflectance Assessments

Absolute reflectance measurements of samples (110 teak clones) were performed using portable NIRQuest512 spectrometer, with a 3.18 mm diameter optical fiber (QR600-7-VIS-125F, Oceanoptics). The reflectance data was extracted using Oceanview Spectroscopy software (ASD Inc., Boulder, CO, USA). Additionally, a set of spectral reflectance indices (SRIs) were calculated and used as predictors in a PLS model (described below). For this, a literature search for spectral reflectance indices (SRIs) was carried out in the Index DataBase [37] for the range of 895–2521 nm, which are listed in Table S1.

### 3.5. Partial Least Squares Regression

The partial least squares regression (PLSR) was applied for model calibration using the R Statistical software version 4.2.1 with the package pls [32,38]. Calibration models were developed with a leave-one-out cross-validation (LOOCV) method. The data sets were divided using a stratified random sampling procedure in order to obtain the training (n = 86) and validation (n = 24) data sets. The PLS components used in each model were selected using a permutation approach, which basically tests whether adding a new component is beneficial to the model [39]. The validation results were also verified using the root mean squared error of prediction of the cross-validation (RMSEC).

## 4. Conclusions

The present study highlights the potential of using spectral reflectance data obtained through near-infrared spectroscopy (NIRS) to predict the magnitude of damage caused by *C. fimbriata* in a diverse panel of teak clones. The results of the study suggest that certain teak clones are relatively resistant to the fungus and could be selected for breeding programs or conservation efforts to improve the sustainability of teak plantations. The study also validates the hypothesis that NIRS-generated data paired with PLSR can effectively screen the magnitude of damage caused by the fungus in teak clones. These findings have important implications for the development of teak genotypes that are resistant to *C. fimbriata*, which will help ensure the long-term sustainability of teak plantations. Overall, the study provides valuable information that could be used to guide future breeding and conservation efforts for teak, a highly valued species with adaptability to a wide range of climatic conditions and soil variations.

## Figures and Tables

**Table 1:** Summary of statistical analysis for growth-related traits and phenotypic traits related to the magnitude of damage caused by the pathogen *Ceratocystis fimbriata* in a diverse panel of *Tectona grandis*.

Source of Variation	Plant Height	Stem Basal Diameter	Apex Diameter	Lesion Length	Lesion Area	Severity
Genotype (G)	***	***	***	***	***	***
Block (B)	NS	***	***	***	***	***
G x B interaction	*	***	NS	NS	**	NS
Mean	30.04 cm	13.04 mm	7.66 mm	7.56 cm	1.47 cm^2^	26.13%
R^2^	0.64	0.60	0.49	0.45	0.55	0.47
Broad-sense heritability (H^2^)	0.63	0.30	0.33	0.16	0.34	0.15

R^2^: Coefficient of determination of the general linear model. H^2^: Broad-sense heritability was estimated using a mixed modelling approach. *, **, ***: significant at the 0.05, 0.01, and 0.01 probability level, respectively.

**Table 2:** Summary of phenotypic traits related to the magnitude of damage caused by the pathogen *Ceratocystis fimbriata* in *Tectona grandis* samples used in this study.

Trait	Samples (Number)	Mean	Standard Deviation	Minimum	Maximum
Lesion area (calibration)	84	1.38 cm^2^	0.71	0.40 cm^2^	3.72 cm^2^
Lesion area (validation)	26	1.42 cm^2^	0.83	0.49 cm^2^	5.43 cm^2^
Lesion length (calibration)	84	8.54 cm	3.96	1.20 cm	18.63 cm
Lesion length (validation)	26	7.79 cm	3.19	3.07 cm	14.40 cm
Severity (calibration)	84	28.88%	12.93	5.59%	57.70%
Severity (validation)	26	27.92%	11.98	9.40%	57.70%

**Table 3:** Model statistics for predicting traits related to the magnitude of damage caused by *Ceratocystis fimbriata* in *Tectona grandis* clones, using NIRS (near-infrared spectroscopy) and SRI (spectral reflectance indexes) data.

Trait	R^2^ (Cal)	SECV (Cal)	R^2^ (Val)	SEP (Val)	RPD	Terms^3^
	Predictions based on NIRS data					
Lesion area	0.907	0.041	0.894	0.042	3.1	2
Lesion length	0.916	0.043	0.883	0.043	2.7	3
Severity	0.903	0.042	0.893	0.043	2.8	4
	Predictions based on SRI					
Lesion area	0.832	0.037	0.783	0.039	2.1	6
Lesion length	0.772	0.039	0.745	0.038	2.7	4
Severity	0.801	0.034	0.771	0.032	2.2	4

(cal): calibration. (val): validation. SECV: standard error of calibration. SEP: standard error of prediction. SECV and SEP are in units of cm^2^, cm, and %, for lesion area, lesion length, and severity, respectively. RPD: ratio of standard error of prediction to standard deviation. Terms: number of terms used in the model selected for cross-validation.

## Data Availability

Data Availability Statements are available in section “MDPI Research Data Policies” at https://www.mdpi.com/ethics (accessed on 20 May 2023).

## Supplementary Materials

The following supporting information can be downloaded at: https://www.mdpi.com/article/10.3390/plants12142734/s1, Table S1. Spectral Reflectance Indices (SRIs) calculated in teak (*Tectona grandis*) clones [40,41,42,43,44,45,46,47,48,49,50,51,52,53,54,55,56,57,58].
