# Supplementary material for: Application of Near-Infrared Reflectance Spectroscopy for Predicting Damage Severity in a Diverse Panel of Tectona grandis Caused by Ceratocystis fimbriata
Source: Plants (Basel). 2023 Jul 23;12(14):2734. doi: 10.3390/plants12142734 (PMC10386596; doi:10.3390/plants12142734)
Supplement: Supplementary file 1 [file plants-12-02734-s001.zip › plants-2457620-supplementary.pdf]

Supplementary Material

Table S1. Spectral Reflectance Indices (SRIs) calculated in teak (*Tectona grandis*) clones.

SRIs	Name	Formula	Reference
CAI	Cellulose absorption Index	$100 \times (0.5 \times (R_{2030} + R_{2210}) - R_{2100})$	40
CAI	Cellulose absorption Index 2	$0.5 \times (R_{2020} + R_{2220}) - R_{2100}$	41
DAI	Difference 1725/970 Difference LAI	$R_{1725} - R_{970}$	42
NDWI-Hyp	Normalized Difference 1070/1200 NDWI-Hyperion	$\frac{R_{1070} - R_{1200}}{R_{1070} + R_{1200}}$	43
ND1080/1180	Normalized Difference 1080/1180	$\frac{R_{1080} - R_{1180}}{R_{1080} + R_{1180}}$	44
ND1080/1260	Normalized Difference 1080/1260	$\frac{R_{1080} - R_{1260}}{R_{1080} + R_{1260}}$	44
ND1080/1450	Normalized Difference 1080/1450	$\frac{R_{1080} - R_{1450}}{R_{1080} + R_{1450}}$	44
ND1080/1675	Normalized Difference 1080/1675	$\frac{R_{1080} - R_{1675}}{R_{1080} + R_{1675}}$	44
ND1080/1260	Normalized Difference 1080/2170	$\frac{R_{1080} - R_{2170}}{R_{1080} + R_{2170}}$	44
LWVI2	Difference 1094/ 1205 Leaf water VI 2	$\frac{R_{1094} - R_{1205}}{R_{1094} + R_{1205}}$	45
ND1180/1450	Normalized Difference 1080/1450	$\frac{R_{1080} - R_{1450}}{R_{1080} + R_{1450}}$	44
ND1180/1675	Normalized Difference 1080/1675	$\frac{R_{1080} - R_{1675}}{R_{1080} + R_{1675}}$	44
ND1180/2170	Normalized Difference 1080/2170	$\frac{R_{1080} - R_{2170}}{R_{1080} + R_{2170}}$	44
ND1260/1450	Normalized Difference 1260/1450	$\frac{R_{1260} - R_{1450}}{R_{1260} + R_{1450}}$	44
ND1260/1675	Normalized Difference 1260/1675	$\frac{R_{1260} - R_{1675}}{R_{1260} + R_{1675}}$	44
ND1260/2170	Normalized Difference 1260/2170	$\frac{R_{1260} - R_{1675}}{R_{1260} + R_{2170}}$	44
NDBleaf	Normalized Difference leaf canopy biomass	$\frac{R_{2160} - R_{1540}}{R_{2160} + R_{1540}}$	46
NDlma	Normalized Difference leaf mass per area	$\frac{R_{2260} - R_{1490}}{R_{2260} + R_{1490}}$	42
ND960/1180	Normalized Difference 960/1180	$\frac{R_{960} - R_{1180}}{R_{960} + R_{1180}}$	44
ND960/1260	Normalized Difference 960/1260	$\frac{R_{960} - R_{1260}}{R_{960} + R_{1260}}$	44

ND960/1450	Normalized Difference 960/1450	$\frac{R_{960} - R_{1450}}{R_{960} + R_{1450}}$	44
ND960/1675	Normalized Difference 960/1675	$\frac{R_{960} - R_{1675}}{R_{960} + R_{1675}}$	44
ND960/2170	Normalized Difference 960/2170	$\frac{R_{960} - R_{2170}}{R_{960} + R_{2170}}$	44
NDLI	Normalized Difference Lignin Index	$\frac{\log(\frac{1}{R_{1754}}) - \log(\frac{1}{R_{1680}})}{\log\left(\frac{1}{R_{1754}}\right) + \log(\frac{1}{R_{1680}})}$	47
NDNI	Normalized Difference Nitrogen Index	$\frac{\log(\frac{1}{R_{1510}}) - \log(\frac{1}{R_{1680}})}{\log\left(\frac{1}{R_{1510}}\right) + \log(\frac{1}{R_{1680}})}$	47
RVIhyp	Simple Ratio 1058/1148	$\frac{R_{1058}}{R_{1148}}$	48
SR1080/1180	Simple Ratio 1080/1180	$\frac{R_{1080}}{R_{1180}}$	44
SR1080/1260	Simple Ratio 1080/1260	$\frac{R_{1080}}{R_{1260}}$	44
SR1080/1450	Simple Ratio 1080/1450	$\frac{R_{1080}}{R_{1450}}$	44
SR1080/1675	Simple Ratio 1080/1675	$\frac{R_{1080}}{R_{1675}}$	44
SR1080/2170	Simple Ratio 1080/2170	$\frac{R_{1080}}{R_{2170}}$	44
SR1180/1080	Simple Ratio 1180/1080	$\frac{R_{1180}}{R_{1080}}$	44
SR1180/1450	Simple Ratio 1180/1450	$\frac{R_{1180}}{R_{1450}}$	44
SR1180/1675	Simple Ratio 1180/1675	$\frac{R_{1180}}{R_{1675}}$	44
SR1180/2170	Simple Ratio 1180/2170	$\frac{R_{1180}}{R_{2170}}$	44
WC	Simple Ratio 1193/1126 Water content	$\frac{R_{1193}}{R_{1126}}$	49
LAIDI	Simple Ratio 1250/1050 Leaf area determining index	$\frac{R_{1250}}{R_{1050}}$	42
SR1260/1080	Simple Ratio 1260/1080	$\frac{R_{1260}}{R_{1080}}$	44
SR1260/1450	Simple Ratio 1260/1450	$\frac{R_{1260}}{R_{1450}}$	44
SR1260/1675	Simple Ratio 1260/1675	$\frac{R_{1260}}{R_{1675}}$	44
SR1260/2170	Simple Ratio 1260/2170	$\frac{R_{1260}}{R_{2170}}$	44
SR1450/1080	Simple Ratio 1450/1080	$\frac{R_{1450}}{R_{1080}}$	44
SR1450/1180	Simple Ratio 1450/1180	$\frac{R_{1450}}{R_{1180}}$	44
SR1450/1260	Simple Ratio 1450/1260	$\frac{R_{1450}}{R_{1260}}$	44

SR1450/960	Simple Ratio 1450/960	$\frac{R_{1450}}{R_{960}}$	44
TM5/TM7	Simple Ratio 1650/2218	$\frac{R_{1650}}{R_{2218}}$	50
SR1675/1080	Simple Ratio 1675/1080	$\frac{R_{1675}}{R_{1080}}$	44
SR1675/1180	Simple Ratio 1675/1180	$\frac{R_{1675}}{R_{1180}}$	44
SR1675/1260	Simple Ratio 1675/1260	$\frac{R_{1675}}{R_{1260}}$	44
SR1675/960	Simple Ratio 1675/960	$\frac{R_{1675}}{R_{960}}$	44
SR2170/1080	Simple Ratio 2170/1080	$\frac{R_{2170}}{R_{1080}}$	44
SR2170/1180	Simple Ratio 2170/1180	$\frac{R_{2170}}{R_{1180}}$	44
SR2170/1260	Simple Ratio 2170/1260	$\frac{R_{2170}}{R_{1260}}$	44
SR2170/960	Simple Ratio 2170/960	$\frac{R_{2170}}{R_{960}}$	44
WBI3	Simple Ratio 950/900 Water band index	$\frac{R_{950}}{R_{900}}$	51
WBI4	Simple Ratio 895/972 Water band index 4	$\frac{R_{895}}{R_{972}}$	52
SR960/1180	Simple Ratio 960/1180	$\frac{R_{960}}{R_{1180}}$	44
SR960/1260	Simple Ratio 960/1260	$\frac{R_{960}}{R_{1260}}$	44
SR960/1450	Simple Ratio 960/1450	$\frac{R_{960}}{R_{1450}}$	44
SR960/1675	Simple Ratio 960/1675	$\frac{R_{960}}{R_{1675}}$	44
SR960/2170	Simple Ratio 960/2170	$\frac{R_{960}}{R_{2170}}$	44
PWI	Plant Water Index, Water Band Index (WBI), Water Index (WI)	$\frac{R_{970}}{R_{900}}$	53
WBI	Simple Ratio 970/902 Water band index	$\frac{R_{970}}{R_{902}}$	54
Single Band 1020	SB1020	R_{1020}	55
Single Band 1040	SB1040	R_{1040}	55
Single Band 1120	SB1120	R_{1120}	55
Single Band 1200	SB1200	R_{1200}	55
Single Band 1400	SB1400	R_{1400}	55
Single Band 1420	SB1420	R_{1420}	55

Single Band 1450	SB1450	R_{1450}	55
Single Band 1490	SB1490	R_{1490}	55
Single Band 1510	SB1510	R_{1510}	55
Single Band 1530	SB1530	R_{1530}	55
Single Band 1540	SB1540	R_{1540}	55
Single Band 1580	SB1580	R_{1580}	55
Single Band 1690	SB1690	R_{1690}	55
Single Band 1780	SB1780	R_{1780}	55
Single Band 1788	SB1788	R_{1788}	55
Single Band 1820	SB1820	R_{1820}	55
Single Band 1900	SB1900	R_{1900}	55
Single Band 1940	SB1940	R_{1940}	55
Single Band 1960	SB1960	R_{1960}	55
Single Band 1980	SB1980	R_{1980}	55
Single Band 2000	SB2000	R_{2000}	55
Single Band 2060	SB2060	R_{2060}	55
Single Band 2080	SB2080	R_{2080}	55
Single Band 2100	SB2100	R_{2100}	55
Single Band 2130	SB2130	R_{2130}	55
Single Band 2180	SB2180	R_{2180}	55
Single Band 2218	SB2218	R_{2218}	55
Single Band 2240	SB2240	R_{2240}	55

Single Band 2250	SB2250	R_{2250}	55
Single Band 2270	SB2270	R_{2270}	55
Single Band 2280	SB2280	R_{2280}	55
Single Band 2300	SB2300	R_{2300}	55
Single Band 2310	SB2310	R_{2310}	55
Single Band 2320	SB2320	R_{2320}	55
Single Band 2340	SB2340	R_{2340}	55
Single Band 2350	SB2350	R_{2350}	55
Single Band 910	SB910	R_{910}	55
Single Band 930	SB930	R_{930}	55
Single Band 970	SB970	R_{970}	55
Single Band 990	SB990	R_{990}	55
sLAIDI	sLAIDI	$S\frac{R_{1050} - R_{1250}}{R_{1050} + R_{1250}}$	56
SWIRFI	Clay index, SWIR Fine particles Index	${R_{2209}}^{3} \times \frac{{R_{2133}}^{2}}{R_{2225}}$	57
SWIRVI	Shortwave-infrared	$37.72 \times (R_{2210} - R_{2090}) + 26.27\,(R_{2280} - R_{2090}) + 0.57$	58
SWIRLI	Shortwave-infrared	$3.87 \times (R_{2210} - R_{2090}) - 27.51\,(R_{2280} - R_{2090}) - 0.2$	58
SWIRSI	Shortwave-infrared	$-41.59 \times (R_{2210} - R_{2090}) + 1.24\,(R_{2280} - R_{2090}) + 0.64$	58
